# Extent and Distribution of Linkage Disequilibrium in the Old Order Amish

**DOI:** 10.1002/gepi.20444

**Published:** 2009-08-20

**Authors:** Cristopher V Van Hout, Albert M Levin, Evadnie Rampersaud, Haiqing Shen, Jeffrey R O'Connell, Braxton D Mitchell, Alan R Shuldiner, Julie A Douglas

**Affiliations:** 1 Department of Human Genetics, University of Michigan School of MedicineAnn Arbor, Michigan; 2 Department of Medicine, University of Maryland School of MedicineBaltimore, Maryland; 3 Geriatric Research and Education Clinical Center, Veterans Administration Medical CenterBaltimore, Maryland

**Keywords:** single nucleotide polymorphism, population genetics, human genetics, founder population, linkage disequilibrium, haplotypes

## Abstract

Knowledge of the extent and distribution of linkage disequilibrium (LD) is critical to the design and interpretation of gene mapping studies. Because the demographic history of each population varies and is often not accurately known, it is necessary to empirically evaluate LD on a population-specific basis. Here we present the first genome-wide survey of LD in the Old Order Amish (OOA) of Lancaster County Pennsylvania, a closed population derived from a modest number of founders. Specifically, we present a comparison of LD between OOA individuals and US Utah participants in the International HapMap project (abbreviated CEU) using a high-density single nucleotide polymorphism (SNP) map. Overall, the allele (and haplotype) frequency distributions and LD profiles were remarkably similar between these two populations. For example, the median absolute allele frequency difference for autosomal SNPs was 0.05, with an inter-quartile range of 0.02–0.09, and for autosomal SNPs 10–20 kb apart with common alleles (minor allele frequency≥0.05), the LD measure *r*^2^ was at least 0.8 for 15 and 14% of SNP pairs in the OOA and CEU, respectively. Moreover, tag SNPs selected from the HapMap CEU sample captured a substantial portion of the common variation in the OOA (∼88%) at *r*^2^≥0.8. These results suggest that the OOA and CEU may share similar LD profiles for other common but untyped SNPs. Thus, in the context of the common variant-common disease hypothesis, genetic variants discovered in gene mapping studies in the OOA may generalize to other populations.

## INTRODUCTION

Many genetic studies of complex traits and diseases are being conducted in population isolates, including the Old Order Amish (OOA) of Lancaster County Pennsylvania [Ginns et al., 1998; Hsueh et al., 2000; Mitchell et al., 2001, 2008; Streeten et al., 2006; Post et al., 2007; Douglas et al., 2008; Wang et al., 2009]. Whether results from these studies will generalize to other populations is dependent (in part) on the similarity of allele frequencies and patterns of linkage disequilibrium (LD) between populations. To inform future genetic studies of the OOA and facilitate comparisons of findings with other populations, we conducted the first genome-wide survey of LD in the OOA and compared our findings to the International HapMap project [Frazer et al., 2007].

Most of the present-day OOA of Lancaster County are the descendants of approximately 200 individuals [Cross, 1976] from central western Europe who immigrated to the United States in the early eighteenth century [McKusick et al., 1964]. Although recent data indicate that the differences in LD between isolated and cosmopolitan populations for common alleles are modest [Bonnen et al., 2006; Service et al., 2006], the uncertain but unique demographic history of the OOA necessitates empirical evaluation of LD.

## SUBJECTS AND METHODS

OOA study subjects were recruited and genotyped (*n* = 861) in the course of the Heredity and Phenotype Intervention (HAPI) Heart study [Mitchell et al., 2008], which was designed to identify gene-environment interactions influencing cardiovascular traits. Because many closely related individuals were deliberately ascertained, we used a simulated annealing algorithm [Douglas and Sandefur, 2008] to select a set of minimally related individuals (30 men and 30 women). The median (range) pair-wise kinship coefficient was 0.03 (0.01–0.04) for the set of 60 vs. 0.03 (0.01–0.3) for the entire sample of 861. For comparison with the OOA, we also utilized 30 men and 30 women (or 60 unrelated parents) from a US Utah population with northern and western European ancestry (abbreviated CEU) in the International HapMap project [Frazer et al., 2007].

## GENOTYPING AND QC METHODS

DNA was extracted from whole blood by standard methods as described previously [Mitchell et al., 2008]. The Affymetrix GeneChip® Human Mapping 500K Array Set was used for the comparison of LD patterns in both the OOA and CEU samples. Genotype calls were made using a Bayesian Robust Linear Model with Mahalanobis (BRLMM) distance classifier [Affymetrix, 2006]. Genotype data for the CEU sample and corresponding annotation for the platform, including chromosome and genomic positions for all single nucleotide polymorphisms (SNPs) on the array, were obtained from the Affymetrix website (http://www.affymetrix.com).

Individuals with >5% missing genotypes, and/or for men > 1 % heterozygous genotypes on the X chromosome, were excluded. A subset of autosomal SNPs (2,068), which were selected to have high information content (minor allele frequency (MAF) ≥0.3), low pair-wise LD (maximum *r*^2^ of 0.44), and coverage across all autosomes (average intermarker spacing of 1.3 cm) in the OOA, were used to infer relationships using the maximum likelihood method implemented in Relpair [Epstein et al., 2000]. We excluded individuals who had an inferred relationship that differed from the pedigree relationship with a likelihood ratio greater than 10^6^. Based on these combined criteria, a total of 24 individuals (out of 861) were excluded from further analysis.

SNPs were required to satisfy the following quality control criteria in both samples: (1) ≤5% uncalled genotypes; (2) ≤5 and ≤1 Mendelian inconsistencies in OOA and CEU samples, respectively, using pedigree diagnostics as implemented in PedCheck [O'Connell and Weeks, 1998]; and (3) Hardy Weinberg Equilibrium *P*-value≥10^−6^ by Fisher's exact test [Wigginton et al., 2005] as implemented in Haploview [Barrett et al., 2005]. To assess genotyping accuracy, we used duplicate genotype data for 61 of the 861 OOA subjects for whom data from the Affymetrix Genome-Wide Human SNP Array 6.0 (overlap of 482,235 SNPs with Affymetrix GeneChips Human Mapping 500K Array Set) were also available. Only SNPs with <2 duplicate inconsistencies were retained for analysis. Of the 500,447 genotypes that mapped to a single location in the human genome, 82,404 failed at least one QC measure in at least one sample. Those SNPs were removed, leaving a total of 409,071 autosomal (Table I) and 8,972 X chromosome (Table AI in the Appendix) SNPs. For the SNPs that passed our quality control criteria, the genotype consistency rate among 61 duplicate pairs was 99.4%.

**Table I:** Summary of autosomal SNPs

	OOA	CEU	Overlap
Total genotyped	489,922	489,922	489,922
> 1 duplicate inconsistency^a^	51,459	NA	NA
>5% missing data^b^	50,085	16,896	8,973
Mendelian inconsistencies^b^,^c^	3,188	1,168	202
*P*<10^−6^ for HWE test^d^	379	217	116
Passed QC filter^e^	415,440	472,851	409,071
Passed QC in both OOA and CEU			
Monomorphic^d^	68,869	57,669	52,467
Polymorphic^d^			
MAF≥0.05	297,605	310,704	287,476
MAF≥0.10	256,614	267,149	240,375
MAF≥0.20	182,941	189,133	161,062

OOA, Old Order Amish; CEU, US Utah residents from HapMap; MAF, minor allele frequency; SNPs that failed a QC measure in either sample were excluded from further analysis, and SNPs with MAF≥0.05 passing QC in both samples (*n* = 287,476) were used for LD analysis.

a Based on the 61 OOA individuals who were also genotyped on the Affymetrix 6.0 array; SNPs with more than one duplicated genotype discrepancy were excluded.

b Based on 837 OOA and 90 CEU individuals (30 trios).

c SNPs with >5 and >1 Mendelian inconsistencies in OOA and CEU, respectively.

d Based on 60 unrelated individuals (30 men and 30 women) from each sample.

e SNPs may fail QC in more than one way, so rows do not sum to the subtotal passing QC.

## STATISTICAL ANALYSES

Fisher's exact test was used to compare allele frequency distributions between the OOA and CEU. For common SNPs (MAF≥0.05) on the same chromosome and within 10 Mb of each other, we used the expectation-maximization (EM) algorithm to obtain maximum likelihood estimates of two-SNP haplotype frequencies and measured pair-wise LD by the *r*^2^ and *D*′ statistics [Lewontin, 1964]. Based on common SNPs, we also identified haplotype blocks in the CEU using an extension of the four-gamete rule [Wang et al., 2002] and estimated haplotype frequencies in both the CEU and OOA using the EM algorithm with a partition-ligation method [Qin et al., 2002] for blocks with >10 SNPs as implemented in Haploview [Barrett et al., 2005]. For each sample, we then calculated and compared the effective number of haplotypes in each block, i.e., (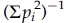, where *p_i_* is the frequency of the ith haplotype in the block. As a measure of redundancy, we identified the number of SNPs (or proxies) that were in strong LD with each SNP at various thresholds of *r*^2^ in each sample. To evaluate the extent to which SNPs selected to tag variation in the CEU capture common variation in the OOA, we selected common tag SNPs in the CEU using the greedy algorithm [Carlson et al., 2004] implemented in Haploview [Barrett et al., 2005] such that every unselected SNP had an *r*^2^≥0.8 with one or more selected SNPs. We then calculated *r*^2^ between the tag SNPs and the remaining “non-tagged” but typed SNPs in the OOA. Unless specified otherwise, all analyses were carried out using a combination of in-house R, Perl, and C programs.

## RESULTS

For the 418,043 SNPs that passed QC, mean heterozygosity was 0.26 and 0.27 for the autosomes in the OOA and CEU, respectively, and 0.23 and 0.24 for the X chromosome. The slightly lower heterozygosity in the OOA reflects the larger number of monomorphic SNPs in the OOA relative to the CEU, e.g., 68,869 vs. 57,669 for the autosomes (Table I). Among all SNPs that were polymorphic in at least one sample, the median absolute allele frequency difference was 0.05 for the autosomes and 0.07 for the X chromosome. At *P*-value<10^−6^, OOA and CEU allele frequencies were significantly different for 799 autosomal and 137 X chromosome SNPs.

The percentage of SNP pairs within 10 Mb of each other and between which strong LD was observed was remarkably similar between the OOA and CEU for the autosomes (Table II) and the X chromosome (Table AII in the Appendix). For example, for autosomal SNPs at an inter-marker distance of <10kb, no evidence of recombination (*D*′ = 1) was observed for 79 and 75% of SNP pairs, perfect LD (*r*^2^ = 1) was observed for 20 and 19% of SNP pairs, and useful LD (*r*^2^≥0.8) was observed for 30 and 29% of SNP pairs in the OOA and CEU, respectively. Based on the CEU sample, we identified 58,097 autosomal haplotype blocks, with a median of three SNPs per block and an inter-quartile range of [3, 4]. Among all autosomal blocks, the median effective number of haplotypes (*n*_e_) was 2.43 and 2.47 in the OOA and CEU, respectively, and the median of the differences in *n*_e_ (CEU minus OOA) per block was 0.04, with an inter-quartile range of −0.2 to 0.3, suggesting modestly greater haplotype diversity in the CEU. Results based on haplotype blocks defined in the OOA did not qualitatively differ from those based on blocks defined in the CEU (data not shown).

**Table II:** Percentage of autosomal SNP pairs^a^ showing no evidence of recombination (*D*′ = 1), perfect LD (*r*^2^ = 1), or where useful LD is observed (*r*^2^ ≥ 0.8)

	*D*′ = 1		*r* = 1		*r*^2^ ≥ 0.8	
			
Inter-SNP distance (kb)	OOA	CEU	OOA	CEU	OOA	CEU
≤10	79	75	20	19	30	29
10–20	60	53	9	7	15	14
20–50	43	34	4	3	9	7
50–100	28	20	1	1	3	2
100–200	20	11	0	0	1	1
200–500	14	7	0	0	0	0
500–1,000	12	6	0	0	0	0
1,000–2,000	11	5	0	0	0	0
2,000–5,000	10	5	0	0	0	0
5,000–10,000	8	5	0	0	0	0

OOA, Old Order Amish (*n* = 60); CEU, US Utah residents from HapMap (*n* = 60).

a Restricted to SNPs with minor allele frequency ≥0.05 in both samples (*n* = 287,476).

Of common autosomal SNPs, 72 and 64% had at least one proxy at *r*^2^≥0.8 and 55 and 44% had at least one perfect proxy (*r*^2^ = 1) in the OOA and CEU, respectively, indicating that fewer independent SNPs are required to represent variation in the OOA relative to the CEU. At *r*^2^≥0.8, 170,979 of 310,704 common SNPs in the CEU were selected as tag SNPs and captured ∼88% of the “non-tagged” SNPs in OOA, suggesting that SNPs selected to tag common variation in the CEU capture much of the same variation in the OOA. SNPs not captured by the CEU tag SNPs tended to be of lower MAF (data not shown). Results for the X chromosome were qualitatively similar.

## DISCUSSION

In general, we found a high degree of similarity in allele frequencies and LD patterns in the OOA and CEU samples. Allele frequencies were not significantly different between the OOA and CEU for > 99% of SNPs. Based on common SNPs, which comprised 74 and 66% of autosomal SNPs in the OOA and CEU, respectively, the distribution and extent of LD were remarkably similar between these two samples. These data are consistent with previous theoretical predictions [Kruglyak, 1999; Pritchard and Przeworski, 2001] and recent empirical data [Bonnen et al., 2006; Service et al., 2006; Navarro et al., 2009; Thompson et al., 2009], all of which point to modest differences in LD between isolated and cosmopolitan populations for common alleles. The situation for rare alleles, however, is likely to be different as has been demonstrated in applications of LD mapping for monogenic diseases and traits.

Demographic and historical information indicate that the OOA were founded relatively recently (∼10–15 generations ago) by a modest number of individuals (several hundred) and then expanded rapidly to a current census population size exceeding 30,000 [Lancaster County Amish, 2002]. Though the precise demographic details are unknown, it is apparent that the number of founders and rate of growth were sufficient and that the subsequent isolation of the OOA was too short for genetic drift and/or recombination to have meaningfully altered the common allele or haplotype frequency spectrum. Our recent study of variation on the Y chromosome supports these observations in that much of the diversity observed in non-isolated populations of similar ancestry is present in the OOA [Pollin et al., 2008]. It appears that inbreeding due to the finite population size of the OOA was also insufficient to meaningfully alter the allele frequency distribution or extent of LD. Based on the 60 OOA individuals included in our analyses, the average inbreeding coefficient *F* [Wright, 1922] was 0.026 (range of 0.0003–0.046), which is too weak to generate substantial differences in LD relative to a non-isolated population [Hill and Robertson, 1968].

Owing to similar allele frequencies and LD patterns in the OOA and CEU, CEU-derived tag SNPs performed well in capturing common variation in the OOA, consistent with previous studies in other samples of European ancestry, including those from isolated populations [Willer et al., 2006; Service et al., 2007]. These results suggest that the OOA and CEU samples may also share similar LD profiles for other common but untyped SNPs. Thus, findings from gene mapping studies in the OOA may generalize to other populations in the context of the common variant-common disease hypothesis.

## APPENDIX

Summary and percentage of X chromosomes are given in Tables AI and AII.

**Table AI:** Summary of X chromosome SNPs

	OOA	CEU	Overlap
Total genotyped	10,525	10,525	10,525
>1 duplicate inconsistency^a^	1,061	NA	NA
>5% missing data^b^	547	461	261
Mendelian inconsistencies^b^,^c^	44	246	10
P<10^−6^ for HWE test^d^	0	0	0
Passed QC filter^e^	9,139	10,064	8,972
Passed QC in both OOA and CEU			
Monomorphic^d^	2,272	1,905	1,805
Polymorphic^d^			
MAF≥0.05	5,763	6,106	5,516
MAF≥0.10	4,971	5,376	4,449
MAF≥0.20	3,571	3,925	2,929

OOA, Old Order Amish; CEU, US Utah residents from HapMap; MAF, minor allele frequency. SNPs that failed a QC measure in either sample were excluded from further analysis, and SNPs with MAF≥0.05 passing QC in both samples (*n*=5,516) were used for LD analysis.

a Based on the 61 OOA individuals who were also genotyped on the Affymetrix 5.0 array; SNPs with more than one duplicated genotype discrepancy were excluded.

b Based on 837 OOA and 90 CEU individuals (30 trios).

c SNPs with >5 and >1 Mendelian inconsistencies in OOA and CEU, respectively.

d Based on 60 unrelated individuals (30 men and 30 women) from each sample.

e SNPs may fail QC in more than one way, so rows do not sum to the subtotal passing QC.

**Table AII:** Percentage of X chromosome SNP pairs^a^ showing no evidence of recombination (*D*′ = 1), perfect LD (*r*^2^ = 1), or where useful LD is observed (*r*^2^≥0.8)

	*D*′ = 1		*r*^2^ = 1		*r*^2^ ≥ 0.8	
			
Inter-SNP distance (kb)	OOA	CEU	OOA	CEU	OOA	CEU
≤10	88	85	39	35	51	49
10–20	72	64	23	19	34	31
20–50	60	48	12	9	21	18
50–100	44	31	6	3	11	10
100–200	31	19	3	1	6	4
200–500	22	11	1	0	2	1
500–1,000	18	7	0	0	0	0
1,000–2,000	17	7	0	0	0	0
2,000–5,000	15	7	0	0	0	0
5,000–10,000	13	7	0	0	0	0

OOA, Old Order Amish (*n* = 60); CEU, US Utah residents from HapMap (*n* = 60).

a Restricted to SNPs with minor allele frequency ≥0.05 in both samples (*n* = 5,516).
